# Comparative efficacy of seven nonpharmacological interventions on global cognition in older adults with and without mild cognitive impairment: a network meta-analysis of randomized controlled trials

**DOI:** 10.1038/s41598-024-58232-2

**Published:** 2024-04-10

**Authors:** Ji-Woo Seok, Gahye Kim, Jaeuk U. Kim

**Affiliations:** 1 Digital Health Research Division, Korea Institute of Oriental Medicine, 1672, Yuseong-daero, Yuseong-gu, Daejeon, 34054 Republic of Korea; 2 KM Convergence Science, University of Science and Technology, Daejeon, South Korea

**Keywords:** Network meta-analysis, Nonpharmacological interventions, Global cognition, Older adults, Mind–body exercise, Intervention mechanism, Geriatrics, Therapeutics

## Abstract

To maintain current cognitive function and access greater cognitive reserves, nonpharmacological interventions may be a viable alternative for older adults with or without cognitive impairment. This study aimed to compare different nonpharmacological interventions for enhancing global cognition, including mind–body exercise, physical exercise, non-invasive brain stimulation, cognitive training intervention (CTI), acutherapy (ACU), meditation, and music therapy, by applying a network meta-analysis (NMA). Sixty-one randomized controlled trials evaluating the efficacy of interventions on global cognition in older adults with or without mild cognitive decline were selected. An NMA was conducted to compare the efficacy of different nonpharmacological interventions. The NMA revealed that mind–body exercise (standardized mean difference, 1.384; 95% confidence interval, 0.777–1.992); ACU (1.283; 0.478–2.088); meditation (0.910; 0.097–1.724); non-invasive brain stimulation (1.242; 0.254–2.230); CTI (1.269; 0.736–1.802); and physical exercise (0.977; 0.212–1.742), showed positive effects compared to passive controls. There were no significant differences between the efficacies of other interventions. Nonpharmacological interventions may potentially enhance and maintain global cognition through various pathways, such as memorizing movements and enhancing brain plasticity by reducing stress in the older adult population. Additional studies are needed to clarify the impact of other variables, including intervention methods or psychological variables.

## Introduction

The global population is experiencing rapid aging. The World Health Organization (WHO) predicts that the proportion of older adults (aged > 60 years) will nearly double, from 12 to 22%, between 2015 and 2050^1^. With an increasingly aging population, cognitive deterioration has become a widespread phenomenon^2^. Current estimates indicate that almost 47 million people worldwide have dementia, with an expected increase to 75 million by 2030^3^. This disease imposes a significant economic burden on society, with the total global societal cost of dementia estimated at US$ 818 billion in 2015 and projected to reach US$ 1 trillion annually by 2030^4^.

To maintain current cognitive function and access greater cognitive reserves, nonpharmacological interventions could serve as a viable alternative to pharmacological interventions for older adults with or without cognitive impairment. Nonpharmacological interventions are likely to be more affordable to develop and implement than pharmacological treatments, while also producing minimal side effects, even when lacking significant efficacy^5^.

Substantial evidence has accumulated from neuroimaging studies showing that nonpharmacological intervention is as effective as pharmacological intervention among older adults^6,7^. Therefore, nonpharmacological interventions, including primary and secondary prevention programs, can be applied in older adults in the early stages of cognitive decline^8^.

Given the benefits of nonpharmacological interventions, recent research has focused on their efficacy, including cognitive training intervention (CTI)^9,10^, music therapy^11,12^, acutherapy (ACU), such as acupuncture and acupressure,^13,14^, meditation^15–17^, physical exercise^18,19^, mind–body exercise^15,20^, and non-invasive brain stimulation^21–23^, for preventing cognitive decline in older adults with or without cognitive impairment. Review and meta-analysis studies of these interventions have demonstrated their effectiveness in maintaining or improving cognitive abilities.

A recent meta-analysis of prospective studies including patients with neurodegenerative diseases reported a small-to-moderate efficacy of CTI when compared to control conditions in global cognitive function in addition to various cognitive domains including, memory, and executive function^10,24^. Musical interventions can help prevent neurodegeneration^25,26^. One meta-analysis demonstrated the beneficial but minimal efficacy of music therapy on global cognition in patients with dementia^27^. A previous meta-analysis demonstrated that ACU intervention was more effective than Western medication for improving global cognition in individuals with mild cognitive impairment (MCI)^13,28,29^. The results of another meta-analysis indicated that non-invasive brain stimulation (transcranial magnetic stimulation [TMS] and transcranial direct current stimulation [tDCS]) has a positive influence, with a small to moderate effect size, in ameliorating cognitive decline in healthy older adults and patients with Alzheimer’s disease (AD)^22,23,30^.

Additionally, a meta-analysis reported that physical exercise was associated with a 28% reduction in incident dementia^31^. Furthermore, a growing number of meta-analyses have suggested that mind–body exercise may improve cognitive function in older adults^15,20,32^. Moreover, previous research has suggested that meditation may contribute to cognitive well-being and emotional balance by enhancing brain regions associated with interoception and attention^33,34^.

These studies suggested that nonpharmacological intervention may enhance cognitive function by increasing the levels of growth factors, modulating inflammatory cytokines, oxidative stress, and autophagy, and attenuating amyloid-beta pathology^35–38^.

While these studies provide an overview of the efficacy and safety of nonpharmacological interventions for global cognition in older patients, they predominantly compare only two interventions (i.e., control condition vs. nonpharmacological intervention), which allows for traditional pairwise meta-analysis. Consequently, a significant knowledge gap exists regarding the relative efficacy of each intervention for cognitive decline. To address this gap, we conducted a network meta-analysis (NMA) to compare the efficacy of several interventions^39^. The NMA for Pharmacoeconomics and Outcome Research strongly recommends comparing the efficacy of different treatment modalities, as endorsed by the International Society for Pharmacoeconomics and Outcome Research (ISPOR)^40^.

Recently, NMAs have compared various nonpharmacological interventions, including physical exercise, music therapy, CTI, and nutritional therapy, in older patients with MCI or AD^41–43^. They found that physical exercise or cognitive stimulation had more significant positive effects on global cognition compared to the other interventions. However, because these studies were conducted exclusively in patients with cognitive impairment, the results primarily pertained to the effectiveness of cognitive rehabilitation rather than cognitive improvement or maintenance. To date, no NMA has been conducted on older adults with minimal or no cognitive impairment to compare the effects of all interventions on global cognition.

Therefore, we performed an NMA of randomized controlled trials to examine the relative effectiveness of seven interventions—CTI, music therapy, non-invasive brain stimulation, ACU intervention, meditation, physical exercise, and mind–body exercise—on the improvement of global cognition among older adults with minimal or no cognitive impairment.

## Methods

### Study protocol registration

This study adhered to the Preferred Reporting Items for Systematic Reviews and Meta-Analyses (PRISMA) guidelines^44^. This NMA was conducted as per the registered protocol in PROSPERO (CRD42023401854). Supplementary Table 1 presents our results in accordance with the PRISMA-NMA checklist.

### Search strategy and study selection

We conducted a systematic literature search of electronic databases including Google Scholar, MEDLINE, PubMed, the Cochrane Central Register of Controlled Trials (CENTRAL), EMBASE, Web of Science, PsycINFO, and ProQuest Dissertations, covering the period from inception to May 2023. The following keywords were applied: (cognition OR global cognition OR cognitive improvement OR cognitive function OR cognitive impairment OR mild cognitive impairment OR MCI) AND (elderly OR elder OR old) AND (randomized OR random OR randomly OR randomization OR randomization OR RCT OR RCTs) AND ((non-pharmacological treatment OR non-pharmacological therapy OR non-pharmacological intervention) OR (Mind–body exercise OR, Baduanjin OR Qi gong OR Tai chi OR Taiji) OR (meditation OR mindfulness OR MBSR) OR (music therapy OR music intervention OR music treatment) OR (non-invasive brain stimulation OR tDCS OR rTMS OR TMS) OR (cognitive training intervention OR cognitive training) OR (acupuncture OR acupressure OR acumassage OR acupoint OR acupoint) OR (exercise OR physical exercise OR fitness OR resistance exercise OR aerobic OR strengthening exercises)). Additionally, we manually reviewed the reference lists of identified publications and relevant articles suggested by meta-analyses and systematic reviews^45^. No restrictions were placed on the language and country of publication as well as the sex or ethnicity of the participants.

### Inclusion and exclusion criteria

To extract data from selected articles, we applied the PICOS (population, intervention, comparison, outcome, and study design) approach.

#### Population

The participants included were healthy older adults or patients with MCI aged 55 years or above. We excluded individuals with moderate-to-severe cognitive impairment (dementia or Parkinson’s disease) or neurological/psychiatric disorders (severe AD, epilepsy, schizophrenia, or multiple sclerosis).

#### Intervention

We included studies that administered structured and conceptualized nonpharmacological interventions, such as CTI, non-invasive brain stimulation, music treatment, mind–body exercise, meditation, and ACU, to healthy older adults and patients with MCI. We excluded studies comparing the efficacy of different treatment approaches within the same intervention category (e.g., Zen meditation vs. Vipassana meditation or tDCS vs. rTMS). Additionally, studies combining multiple interventions were excluded due to difficulties in isolating the efficacy of each intervention from the combined effects (e.g., CTI combined with meditation vs. CTI) (Table 1).

**Table 1:** Nonpharmacologic interventions for cognitive impairment in older adults.

Intervention	Information
Cognitive training intervention (CTI)	CTI is defined as the repeated practice of theoretically driven skills and strategies designed to target cognitive domains such as attention, memory, executive function, and processing speed
Music therapy	Music therapy involves the use of music to alleviate stress and improve self-expression. Music intervention is an intense, multisensory, and motor experience that fosters the creation of new brain connectivity and enhances cerebral plasticity
Acutherapy (ACU)	ACU interventions, including acupuncture, electroacupuncture, and acupressure, have been utilized as mainstays of Oriental medicine. They stimulate acupoints that connect to specific anatomical structures of the peripheral nervous system
Non-invasive brain stimulation	Non-invasive brain stimulation techniques (i.e., transcranial magnetic stimulation [TMS] and transcranial direct current stimulation [tDCS]) are powerful methods for modulating human brain function. They stimulate or alter brain activity at the surface by delivering an electric current
Physical exercise	Exercise is a form of physical activity that involves intentional, organized, and repetitive body movements aimed at enhancing and/or preserving various aspects of physical fitness
Mind–body exercises	Mind–body exercises, including Tai Chi, Qigong, and Baduanjin, consist of gentle exercises characterized by slow-paced, low-intensity, and repeated movements, making them particularly suited to older adults. However, mind–body exercise differs from traditional physical exercise as it places a high cognitive demand and emphasizes cognitive well-being
Meditation	Meditation is a self-regulatory practice that focuses on training attention and awareness to induce integrated physiological changes

#### Comparison

All studies had to include other types of nonpharmacological interventions or control groups. The control group was defined as a passive control group that did not receive any intervention (e.g., waiting list, treatment, or care as usual) or a control group that was provided with other activities (e.g., exercise or sham intervention).

#### Outcome

The studies must have evaluated global cognition^46,47^ using measures such as the Mini-Mental State Examination (MMSE)^48^, the Montreal Cognitive Assessment (MoCA)^49^, the Repeatable Battery for the Assessment of Neuropsychological Status (RBANS)^50^, the Cambridge Cognitive Examination (CAMCOG)^51^, the Mattis Dementia Rating Scale^52^, and the Alzheimer’s Disease Assessment Scale-Cognitive subscale (ADAS-Cog)^53^.

*Study design*: This NMA identified randomized controlled trials (RCTs) that assessed the efficacy of nonpharmacological interventions on global cognition. Case reports and case series were excluded.

### Data extraction

We created a predefined data extraction sheet formed on a pilot extraction with highly related references, and two research assistants extracted the relevant information by applying this template. For data synthesis, a data extraction form included the information such as title, authors, publication year, participants’ characteristics, including age, sex, sample size, and presence of cognitive impairment (i.e., healthy or MCI), nonpharmacological intervention details, types of control conditions, intervention duration, assessments of global cognition, and pre- and post-intervention scores for global cognition. This NMA only included the studies reporting the mean or standard deviation of pre- or post-treatment measurements. If multiple post-intervention scores were reported at different follow-up time points, only the evaluation immediately following the completion of the intervention phase was considered. The extracted data were cross-checked and consensus was reached by discussion; any disagreements were adjudicated by a third party.

### Quality appraisal

Two independent reviewers conducted full-text screening of the included articles and evaluated the quality of individual studies using the Cochrane risk-of-bias tool for RCTs^54^. Quality appraisal considered various bias domains, including selection bias (random sequence generation and allocation concealment), reporting bias (selective reporting), performance bias (blinding of participants and personnel), detection bias (blinding of outcome assessment), attrition bias (incomplete outcome data), and other sources of bias. These items were rated as low, high, or unclear. Quality appraisals were cross-checked, and any disagreements between the reviewers were resolved through discussion or adjudication by a third party.

## Summary measure

### Calculation of effect size

We conducted an NMA that comprised 60 studies utilizing the pretest–posttest control (PPC) design. The PPC design offers a more efficient framework for estimating treatment effects compared to pretest–posttest comparison group or posttest-only control group designs. Therefore, we excluded studies with other study designs from the analyses. Intervention efficacies were evaluated using Cohen’s d effect size. To determine the d value, we applied the effect size estimate suggested by Morris^55^. Effect size was calculated using the mean pre-post change in the intervention group minus the mean pre-post change in the control group, standardized by the pooled pre-intervention standard deviation, as follows:^55,56^

$$d=\frac{\left({M}_{post-I}- {M}_{pre-I}\right)-({M}_{post-c}- {M}_{pre-c})}{{SD}_{pooled}}* {C}_{p}$$

*M*_*post-I*_*, M*_*pre-I*_*, M*_*post-c*_*, and M*_*pre-c*_ are the mean scores of the intervention and control groups for the posttest and pretest, respectively. Additionally, *SD*_*pooled*_* and C*_*p*_ are the pooled standard deviation and the bias correction, respectively.

D values of 0.2–05 are considered small, those of 05–0.8 are considered moderate, and those > 0.8 are considered large^57^.

### Network meta-analysis

As outlined in the study protocol, we categorized seven nonpharmacological interventions and two control conditions as follows: (1) CTI, (2) non-invasive brain stimulation, (3) music therapy, (4) ACU, 5) mind–body exercise, (6) meditation, (7) physical exercise, (8) passive control, and (9) sham intervention. Following categorization, we performed an NMA using a frequentist random-effects model with the net-meta R package (version 8.0)^58^ to compare the efficacy between interventions. The network plot chart displayed nodes representing each intervention and control condition, with lines indicating direct comparisons between two interventions or control conditions. The width of each line was proportional to the number of studies reporting pairwise comparisons^58^.

Heterogeneity between studies was assessed using Q statistics and I^2^, which describe the proportion of total variability in effect estimates across studies attributable to heterogeneity rather than sampling error^58,59^. A *P* value < 0.05 for the Q statistic indicated significant heterogeneity^60^. To rank the interventions based on their efficacy for each outcome, P-scores ranging from 0 to 1 were obtained by estimating the effect sizes of pairwise intervention comparisons using the surface under the cumulative ranking curve (SUCRA)^61^. A higher P-score indicated a more effective intervention, with 0 or 1 representing the worst or best intervention, respectively^58^.

Additionally, we performed a post-hoc subgroup analysis comparing MCI and healthy older adult groups.

### Assessment of inconsistency

To assess the possible sources of inconsistency, we employed two methods: net heat plot and node-splitting analysis. The net heat plot is a matrix visualization method used to identify inconsistencies within the NMA^62^. In this plot, each gray box represents the contribution of the direct estimates to the network estimates, with the size of the box indicating the magnitude of the contribution. The color of the box indicates changes in inconsistency, with blue or warm colors representing an increase or decrease in inconsistency, respectively^62^. Additionally, we conducted a node-splitting analysis to evaluate the inconsistency between direct and indirect estimates in the forest plots of the NMA^63^. To address publication bias, we examined the adjusted funnel plots. Egger’s test was used to test the asymmetry of the funnel plot^64^.

## Results

### Study selection

**Figure 1:**
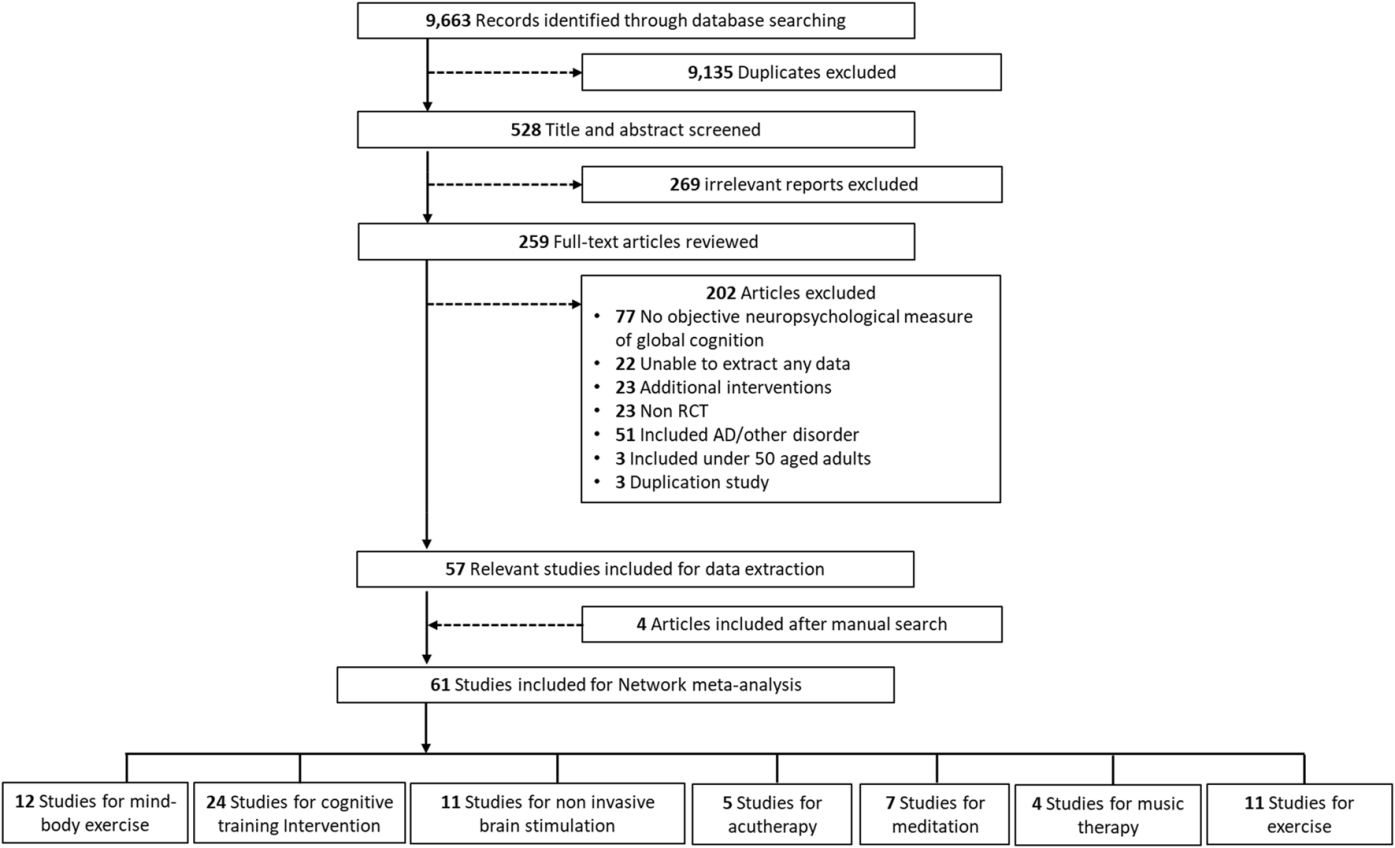
Flow diagram of study selection.

Figure 1 illustrates the flow diagram depicting the selection of the included studies. Initially, our search yielded 9663 papers related to our research terms. Of them, 9135 duplicate and irrelevant papers were excluded during the title/abstract screening. Additionally, 202 papers were omitted because of unavailability of data or the presence of adjunctive treatment. Through manual searching, four additional studies were identified. Ultimately, 61 RCTs were included in the NMA (Fig. 1 and Table 2). Two studies compared individual intervention arms (CTI vs. acupressure and CTI vs. Meditation) (Table 2).

**Table 2:** Studies measuring externalizing symptoms.

Author & year	Participants	Age	Groups	Duration of intervention	Cognitive assessment
Mind–body exercise					
Chen et al. ^70^	MCI	67.55 ± 5.02	Tai Chi (n = 107), Fitness walk (n = 110), No intervention (n = 111)	3 days/week, 60 min/session, 6 months	MoCA
Dechamps et al., 2010	healthy	82.27 ± 9.11	Tai Chi (n = 51), Cognition-action (n = 49), No intervention (n = 60)	4 days/week, 30 min/session, 6 months	MMSE
Hwang et al., 2016	healthy	72.35 ± 8.1	Tai Chi (n = 167), Exercise (n = 167)	1 day/week, 60 min/session, 6 months	MMSE
Lam et al., 2011	MCI	67.56 ± 4.99	Tai Chi (n = 135), Exercise (n = 194)	3 days/week, 30 min/session, 12 months	MMSE, ADAS-Cog
Lam et al., 2012	MCI	77.82 ± 6.47	Tai Chi (n = 92), Exercise (n = 169)	3 days/week, 30 min/session, 6 months	MMSE, ADAS-Cog
Li et al., 2014	healthy	76.04 ± 10.10	Tai Chi (n = 22), No intervention (n = 24)	7 days/week, 12 min/session, 2 months	MMSE
Siu et al., 2018	MCI	60 ≥ years	Tai Chi (n = 80), No intervention (n = 80)	2 days/week, 60 min/session, 4 months	MMSE
Sun et al., 2015	healthy	69.16 ± 5.81	Tai Chi (n = 72), No intervention (n = 66)	2 days/week, 60 min/session, 6 months	MMSE
Tsai et al., 2013	MCI	78.91 ± 7.55	Tai Chi (n = 28), Education (n = 27)	3 days/week, 20–40 min/session, 5 months	MMSE
Yu et al., 2022	healthy	65.90 ± 4.73	Tai Chi (n = 21), Brisk walking (n = 22)	3 days/week, 60 min/session, 10 months	MoCA
Mortimer et al., 2012	healthy	67.77 ± 5.64	Tai Chi (n = 30), No intervention (n = 30), Walking (n = 30)	3 days/week, 50 min/session, 10 months	Mattis Dementia Rating Scale
Lin et al., 2023	healthy	66.52 ± 5.17	Baduanjin (n = 51), Health education program (n = 51)	3 days/week, 60 min/session, 6 months	MoCA
Non-invasive brain stimulation					
Padala et al., 2018	MCI	65.6 ± 9.3	rTMS (n = 8), Sham intervention (n = 8)	5 days/week, 45 min/session, 2 months	MMSE
Yuan et al., 2021	MCI	64.88 ± 4.81	rTMS (n = 12), Sham intervention (n = 12)	5 days/week, 4 months	MoCA
Roque et al., 2021	MCI	66.36 ± 5.12	rTMS (n = 22), Sham intervention (n = 22)	3 days/week, 10 days	MoCA, MMSE
Drumond Marra et al., 2015	MCI	65.16 ± 3.85	rTMS (n = 15), Sham intervention (n = 19)	5 days/week, 10 days	MoCA
Moghadam et al., 2020	healthy	61.63 ± 2.37	tDCS (n = 30), Sham intervention (n = 15)	5 days/week, 2 months	MMSE
Fileccia et al., 2019	MCI	70.65 ± 6.20	tDCS (n = 17), Sham intervention (n = 17)	5 days/week, 20 min/session, 4 months	MMSE
Gomes et al., 2019	MCI	72.3 ± 8.57	tDCS (n = 29), Sham intervention (n = 29)	2 days/week, 30 min/session, 5 months	MMSE
He et al., 2021	MCI	64.44 ± 6.36	tDCS (n = 24), Sham intervention (n = 19)	5 days/week, 20 min/session, 2 months	MMSE, MoCA
Lu et al., 2019	MCI	74.34 ± 6.65	tDCS (n = 69), Sham intervention (n = 64)	3 days/week, 45 min/session, 4 months	ADAS-Cog
Manor et al., 2018	MCI	82 ± 4	tDCS (n = 9), Sham intervention (n = 9)	20 min/session, 2 months	MoCA
Rodrigues et al., 2023	healthy	71.6 ± 7.67	tDCS (n = 14), Sham intervention (n = 14), no intervention (n = 15)	2 days/week, 20 min/session, 2 months	MMSE
Cognitive training intervention					
Kim et al., 2005	MCI, Healthy	79.3 ± 6.07	Cognitive training intervention (n = 15), Movie (n = 15)	3 days/week, 20–30 min/session, 4 months	MMSE
Canete et al., 2019	MCI	72.7 ± 7.65	Cognitive training intervention (n = 8), Kirtan Kriya meditation (n = 7)	1 days/week, 12 min/session, 2 months	MMSE
Wittelsberger et al., 2012	MCI	70.08 ± 13.02	Cognitive training intervention (n = 17), No intervention (n = 10)	2 days/week, 45–60 min/session, 6 months	MMSE
Hughes et al., 2014	MCI	77.35 ± 5.87	Cognitive training intervention (n = 10), Education (n = 10)	1 day/week, 90 min/session, 6 months	CAMCI
Fiatarone Singh et al., 2014	MCI	55 ≥ years	Cognitive training intervention (n = 24), Exercise (n = 27)	3 days/week, 75 min/session, 6 months	ADAS-Cog
Barcelos et al., 2015	Healthy	82.53 ± 9.75	Cognitive training intervention (n = 33), Exercise (n = 27)	3–5 days/week, 45 min/session, 3 months	MMSE
Peng et al., 2019	MCI	68.54 ± 4.24	Cognitive training intervention (n = 70), No intervention (n = 70)	Every 2 weeks for 6 months, 90 min/session	MoCA
Gooding et al., 2016	MCI	75.59 ± 8.75	Cognitive training intervention (n = 31), Internet activity (n = 20)	2 days/week, 60 min/session, 4 months	MMSE
Ciarmiello et al., 2015	MCI	71.59 ± 7.40	Cognitive training intervention (n = 15), Education (n = 15)	2 days/week, 45 min/session, 4 months	MMSE
Djabelkhjr et al., 2017	MCI	76.78 ± 6.72	Cognitive training intervention (n = 9), Internet activity (n = 10)	1 day/week, 90 min/session, 3 months	MMSE
Hagovská et al., 2016	MCI	66.98 ± 5.35	Cognitive training intervention (n = 40), Exercise (n = 38)	2 days/week, 30 min/session, 5 months	MMSE
Han et al., 2017	MCI	74.15 ± 5.75	Cognitive training intervention (n = 19), usual care (n = 22)	2 days/week, 30 min/session, 4 months	MMSE
Savulich et al., 2017	MCI	76.05 ± 7.86	Cognitive training intervention (n = 21), usual care (n = 21)	2 days/week, 60 min/session, 4 months	MMSE
Li et al., 2019	MCI	70.39 ± 7.08	Cognitive training intervention (n = 78), No intervention (n = 63)	3–4 days/week, 120–160 min/session, 6 months	MMSE
Forster et al., 2011	MCI	73.18 ± 7.84	Cognitive training intervention (n = 17), Other activity (n = 19)	1 day/week, 120 min/session, 6 months	ADAS-Cog
Rojas et al., 2013	MCI	74.47 ± 11.27	Cognitive training intervention (n = 15), Other activity (n = 15)	2 days/week, 120 min/session, 6 months	MMSE
Popsti et al., 2018	MCI	68 ± 8.5	Cognitive training intervention (n = 14), No intervention (n = 14)	2 days/week, 60 min/session, 6 months	MMSE
Cavallo et al., 2019	MCI	76.42 ± 3.39	Cognitive training intervention (n = 40), Other activity (n = 40)	3 days/week, 30 min/session, 3 months	MMSE
Lazarou et al., 2019	MCI	73.8 ± 6.43	Cognitive training intervention (n = 6), No intervention (n = 6)	2 days/week, 60 min/session, 4–12 months	MMSE
Thapa et al., 2020	MCI	72.65 ± 5.50	Cognitive training intervention (n = 33), Education (n = 33)	3 days/week, 100 min/session, 2 months	MMSE
Millan-Calenti et al., 2015	Healthy	74.34 ± 6.36	Cognitive training intervention (n = 80), No intervention (n = 62)	7 days/week, 20 min/session, 3 months	MMSE
Nouchi et al., 2012	Healthy	69.09 ± 2.47	Cognitive training intervention (n = 14), Other activity (n = 14)	7 days/week, 15 min/session, 1 month	MMSE
Wang et al., 2011	Healthy	64.2 ± 6.69	Cognitive training intervention (n = 26), No intervention (n = 26)	1 day/week, 45 min/session, 5 weeks	MMSE
Sun et al., 2021	MCI	60 ≥ years	Cognitive training intervention (n = 38), Acupressure (n = 38), No intervention (n = 38)	5 days/week, 6 months	MMSE
Acutherapy					
Fan et al., 2019	Healthy	66.8 ± 5.50	Electroacupuncture (n = 15), No intervention (n = 15)	5 days/week, 25 min/session, 2 months	MoCA
Zhao et al., 2018	Healthy	60–75	Electroacupuncture (n = 30), Sham intervention (n = 30)	1 day/week, 30 min/session	MMSE
Choi et al., 2021	MCI	63.71 ± 7.21	Electroacupuncture (n = 20), Education (n = 19), Sham intervention (n = 20)	2 days/week, 3 months	MoCA
Lei et al., 2015	Healthy	72.06 ± 7	Acupoint massage (n = 34), Education (n = 34)	7 days/week, 1 month	MMSE
Sun et al., 2021	MCI	60 ≥ years	Cognitive training intervention (n = 38), Acupressure (n = 38), No intervention (n = 38)	5 days/week, 6 months	MMSE
Meditation					
Canete et al., 2019	MCI	72.7 ± 7.65	Cognitive training intervention (n = 8), Kirtan Kriya meditation (n = 7)	1 day/week, 12 min/session, 2 months	MMSE
Lavretsky et al., 2013	healthy	60.54 ± 23.09	Meditation (n = 23), Music (n = 16)	5 days/week, 12 min/session, 2 months	MMSE
Newberg et al., 2010	MCI	64.35	Meditation (n = 9), Music (n = 5)	7 days/week, 12 min/session, 2 months	MMSE
Sun et al., 2013	healthy	72.81 ± 7.97	Meditation (n = 37), Education (n = 38)	2 days/week, 30 min/session, 13 months	MMSE
Zheng et al., 2021	healthy	65.82 ± 4.84	Meditation (n = 20), Usual physical activity (n = 20)	3 days/week, 60 min/session, 6 months	MoCA
Klainin-Yobas et al., 2019	MCI	71.35 ± 5.80	Meditation (n = 28), Education (n = 27)	1 day/week, 40 min/session, 6 months	MMSE
Ernst et al., 2008	healthy	85.91	Meditation (n = 9), Other activity (n = 7)	1 day/week, 90 min/session, 2 months	MMSE
Music therapy					
Doi et al., 2017	MCI	76.09 ± 4.76	Music therapy (n = 54), Education (n = 63)	1 day/week, 60 min/session, 10 months	MMSE
Han et al., 2020	MCI	73.13 ± 6.95	Music therapy (n = 12), Sham intervention (n = 12)	2 days/week, 60 min/session, 10 weeks	MMSE
Tai et al., 2015	healthy	80.87 ± 7.80	Music therapy (n = 41), No intervention (n = 19)	5 days/week, 30 min/session, 4 months	MMSE
Biasutti et al., 2019	healthy	83.58 ± 7.16	Music therapy (n = 20), Exercise (n = 25)	2 days/week, 70 min/session, 6 weeks	MMSE
Exercise					
Barcelos et al., 2015	healthy	82.53 ± 9.75	Cognitive training intervention (n = 33), Exercise (n = 27)	3–5 days/week, 45 min/session, 3 months	MMSE
Fiatarone Singh et al., 2014	MCI	55 ≥ years	Cognitive training intervention (n = 24), Exercise (n = 27)	5 days/week, 30 min/session, 6 months	ADAS-Cog
Hagovská et al., 2016	MCI	66.98 ± 5.35	Cognitive training intervention (n = 40), Exercise (n = 38)	2 days/week, 30 min/session, 5 months	MMSE
Chen et al., 2023	MCI	67.55 ± 5.02	Tai Chi (n = 107), Fitness walk (n = 110), No intervention (n = 111)	3 days/week, 60 min/session, 6 months	MoCA
Dechamps et al., 2010	healthy	82.27 ± 9.11	Tai Chi (n = 51), Cognition-action (n = 49), No intervention (n = 60)	4 days/week, 30 min/session, 6 months	MMSE
Hwang et al., 2016	healthy	72.35 ± 8.1	Tai Chi (n = 167), Exercise (n = 167)	1 day/week, 60 min/session, 6 months	MMSE
Lam et al., 2011	MCI	67.56 ± 4.99	Tai Chi (n = 135), Exercise (n = 194)	3 days/week, 30 min/session, 12 months	MMSE, ADAS-Cog
Lam et al., 2012	MCI	77.82 ± 6.47	Tai Chi (n = 92), Exercise (n = 169)	3 days/week, 30 min/session, 6 months	MMSE, ADAS-Cog
Yu et al., 2022	healthy	65.90 ± 4.73	Tai Chi (n = 21), Brisk walking (n = 22)	3 days/week, 60 min/session, 10 months	MoCA
Mortimer et al., 2012	healthy	67.77 ± 5.64	Tai Chi (n = 30), No intervention (n = 30), Walking (n = 30)	3 days/week, 50 min/session, 10 months	Mattis Dementia Rating Scale
Biasutti et al., 2019	healthy	83.58 ± 7.16	Music therapy (n = 20), Exercise (n = 25)	2 days/week, 70 min/session, 6 weeks	MMSE

*ADAS-Cog* Alzheimer’s Disease Assessment Scale-Cognitive subscale, *MCI* Mild Cognitive Impairment, *MMSE* Mini Mental State Examination, *MoCA* Montreal Cognitive Assessment.

### Study characteristics

Table 2 presents the characteristics of the included studies. All included studies were conducted and published between January 2005 and December 2023. A total of 5458 older adults (1626 healthy individuals and 3832 patients with MCI) participated in the 61 RCTs included in the analysis. Among these, 2772 and 2686 participants were randomly assigned to the intervention (i.e., Qigong, music therapy, CTI, non-invasive brain stimulation, ACU, and meditation) and control conditions (passive control, exercise, and sham intervention), respectively. The age range of the participants in the RCTs was 55–89 years, with a mean age of 70.12 years. Three studies did not provide specific age information.

The mean duration of Qigong, non-invasive brain stimulation, CTI, ACU, meditation, and music therapy was 72.73 (SD = 34.72), 40.08 (SD = 24.63), 46.28 (SD = 27.87), 57.33 (SD = 45.18), 50.67 (SD = 34.56), and 40.80 (SD = 25.98) days, respectively. Two studies did not report the duration. The mean session duration for Qigong, non-invasive brain stimulation, CTI, ACU, meditation, and music therapy was 44.23 (SD = 17.27), 29.00 (SD = 10.25), 63.60 (SD = 37.54), 27.50 (SD = 3.54), 30.80 (SD = 20.28), and 85.00 (SD = 65.57) minutes, respectively.

### Network meta-analysis results for global cognition

**Figure 2:**
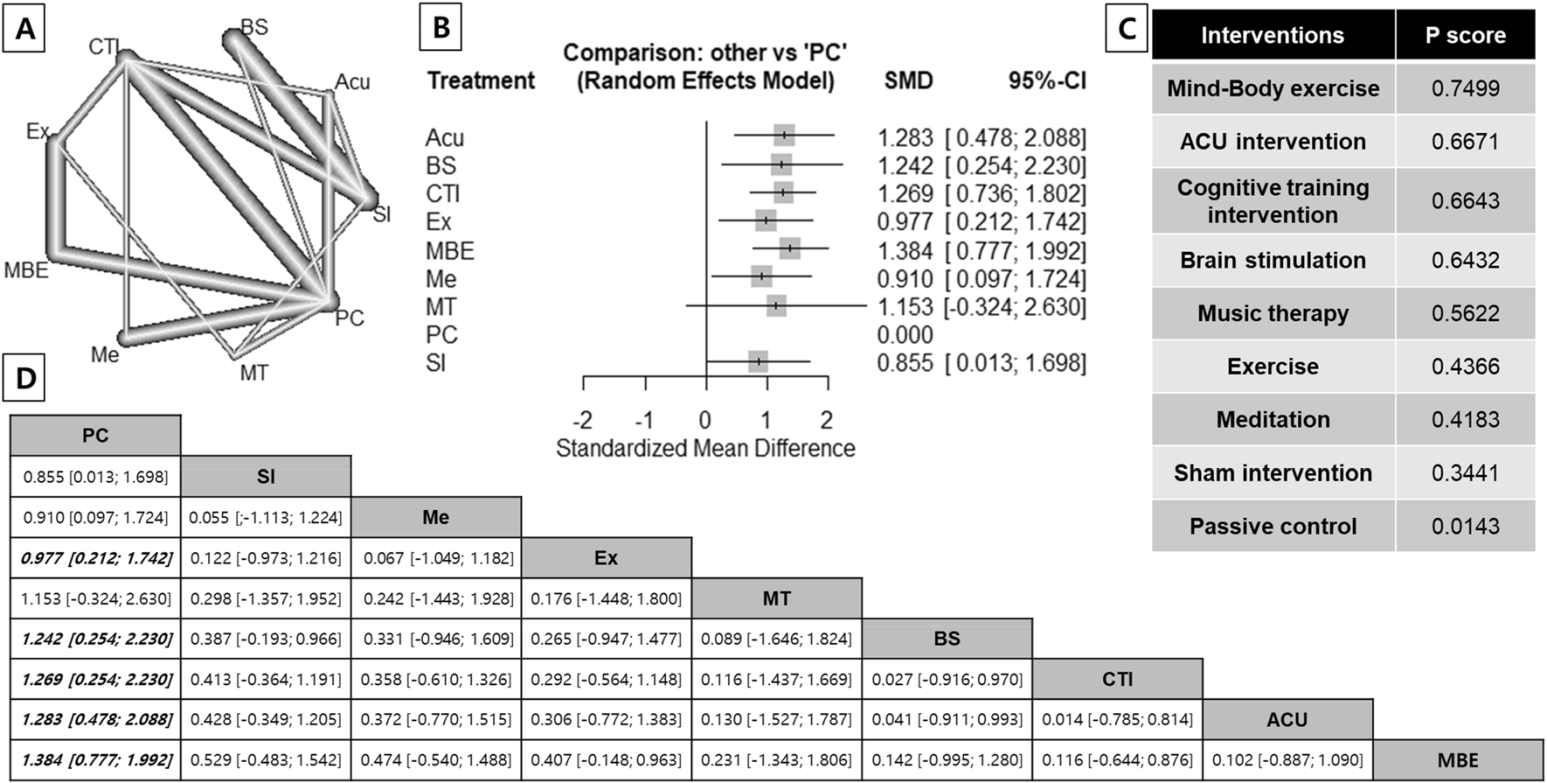
The result of network meta-analysis for global cognition. (**A**) The network graph representing treatment arms included in the network for global cognition; the thickness of the lines shows the number of studies. (**B**) Random effects model forest plot for comparison of each intervention arm vs. passive control. (**C**) Ranking of medications for disruptive symptoms using SUCRA values. (**D**) Comparison of the included interventions: standardized mean differences (95% CI). Each cell represents the effect of the column-defining intervention compared to the row-defining intervention. *BS* Non-invasive brain stimulation, *CI* Confidence interval, *CTI* Cognitive training intervention, *Ex* Exercise, *MBE* Mind–Body exercise, *Me* Meditation, *MT* Music therapy, *PC* Passive control, *SI* Sham intervention, *SUCRA* Surface Under the Cumulative Ranking curve.

Figure 2a displays the network diagram of 60 studies comparing eight intervention groups with passive controls, including mind–body exercise, CTI, non-invasive brain stimulation, ACU, meditation, music therapy, exercise, and sham intervention. All intervention groups had at least one passive control group. The forest plot in Fig. 2b represents the relative efficacy of each intervention compared with passive control for global cognition.

When combined in the NMA, six interventions were significantly more effective than passive control for global cognition: mind–body exercise, with a standardized mean difference (SMD) of 1.384 (95% CI 0.777–1.992); ACU, with 1.283 (95% CI 0.478–2.088); non-invasive brain stimulation, with 1.242 (95% CI 0.254–2.230); CTI, with 1.269 (95% CI 0.736–1.802); exercise, with 0.977 (95% CI 0.212–1.742); and meditation with 0.910 (95% CI 0.097–1.724) (Fig. 2b).

The ranking order of intervention efficacy for global cognition is as follows: mind–body exercise (P score = 0.7499), ACU (P-score = 0.6671), CTI (P-score = 0.6643), brain stimulation (P-score = 0.6432), music therapy (P-score = 0.5622), exercise (P-score = 0.4366), meditation (P-score = 0.4183), and sham intervention (P-score = 0.3441). Passive controls are ranked last in improving global cognition (P-score = 0.0143) (Fig. 2c).

Detailed results of the NMAs with different references are presented in Fig. 2d. Despite having the highest efficacy for global cognition compared to placebo, mind–body exercise did not show significant superiority over other interventions in direct and indirect comparisons. No significant differences in global cognition were observed between the interventions, except in comparison to passive controls (Fig. 2d).

In the results of subgroup analyses, the MCI and healthy groups responded to interventions differently (Supplementary Figure s1). In the MCI group, ACU intervention, CTI, non-invasive brain stimulation, and mind–body exercise were significantly effective compared to the passive control. However, in the healthy control group, mind–body exercise, ACU intervention, CTI, and meditation showed significant efficacy for global cognition.

### Quality assessment

We conducted a quality assurance analysis using Review Manager software version 5.4 (Nordic Cochrane Center, Copenhagen, Denmark). Supplementary Figures S2 and S3 present a summary of the risk of bias across studies and the methodological features of each study.

The network quality analysis for global cognition indicated no evidence of heterogeneity (Q = 39.24, P = 0.9937, tau^2^ = 0.000, I^2^ = 0%). Additionally, no significant discrepancy was found between direct and indirect estimates of global cognition (Supplementary Tables S2 and S3).

The node-splitting method, which evaluates the incoherence in all pairs of direct and indirect estimates, revealed no significant inconsistency in the direct and indirect estimates (Supplementary Figure S4). Net heat plots were generated to visualize the consistency pattern in each comparison and detect important inconsistencies expressed as hot spots (red squares) (Supplementary Figure S5).

The risk of publication bias in the NMA was evaluated using the generated funnel plots (Supplementary Figure S6). Most studies exhibited a normal distribution, and no significant asymmetric pattern was observed in the funnel plots. Consequently, our study did not provide evidence of publication bias.

## Discussion

This is the first RCT NMA to assess the individual efficacy of nonpharmacological interventions on global cognition in older adults with minimal or no cognitive decline. The NMA results showed that mind–body exercise, ACU intervention, meditation, non-invasive brain stimulation, CTI, and physical exercise had significant effects on improving global cognition compared to passive control conditions, with large effect sizes. The ranking order of intervention efficacy for global cognition is as follows: mind–body exercise, ACU, CTI, brain stimulation, music therapy, exercise, and meditation. However, no significant differences were found between the interventions, including the sham intervention (Fig. 2d).

Previous NMAs that focused on different interventions in older patients with AD or MCI also demonstrated the positive effect of physical exercise, ranking it higher than other interventions^41–43^. Consistent with these findings, our study showed that mind–body exercise was ranked first for improving global cognition (P-score = 0.7984). Mind–body exercises involve specific body postures, controlled breathing techniques, and mental concentration. Although there are several types of mind–body exercises (i.e., Qigong, Tai Chi, and Baduanjin), all consist of physical movement, breathing, and meditative exercises^65^. While the relationship between mind–body exercise and global cognition has not been systematically investigated, previous studies have suggested that the cognitive improvement observed after mind–body exercise may be attributed to cognitive stimulation and changes in stress-related physiological systems^66,67^. Compared to traditional exercises, mind–body exercises involve factors that provide additional cognitive stimulation^66^. Mind–body exercises require various higher-order cognitive functions beyond just memory training, such as attention, visual-spatial ability, perceptual speed, multitasking, and planning, in order to maintain postural stability^66^.

Both meditation and mind–body exercise have down-regulating effects on the sympathetic nervous system and the hypothalamic–pituitary–adrenal (HPA) axis activities triggered by stress^68^. Activation of the HPA axis in response to stress leads to increased secretion of cortisone, which can disrupt synaptic plasticity, damage hippocampal dendrites, and reduce neurogenesis in the adult brain, ultimately resulting in cognitive impairment^69^.

Large effect sizes (SMD > 0.8) were observed for ACU intervention, meditation, CTI, and non-invasive brain stimulation in improving global cognition. Although ACU interventions have not been extensively studied in meta-analyses of acupuncture, there is growing evidence supporting their cognitive improvement effects. ACU intervention at the acupoints “Baihui,” “Dazhui,” “Shenshu,” and “Zusanli” improved cognitive function, especially learning and memory, by repairing synaptic damage in patients with cerebral ischemia, and exerted neuroprotective effects by modulating the expression of *CREB*, *BDNF*, *BCL2*, and *BAX* genes^70,71^.

CTI has been validated and proven effective in improving and maintaining cognition and preventing dementia^72^. The prevailing hypothesis suggests that repeated practice with a set of tasks targeting specific cognitive domains (e.g., attention, memory, language, and executive function) may enhance functioning in those domains, leading to improvements in global cognition^72^. CTIs have the potential to activate neuroplasticity and increase cognitive reserve, which refers to the brain’s capacity to mitigate losses resulting from brain injury or degeneration^73^.

Non-invasive brain stimulation is an emerging tool for rehabilitating cognition in patients with neurodegenerative diseases, although its therapeutic mechanisms are not yet fully understood^74^. It has been hypothesized that high-frequency electric current stimulation induces various functional changes, including physiological (i.e., modulation of cortical excitability) and metabolic effects (i.e., enhanced neurotransmitter release), which in turn promote structural reorganization of the brain through neuroreparative processes^75–77^.

Music therapy is widely employed in clinical practice to benefit patients with dementia^25^. While the effect size is small (SMD < 0.3), numerous reviews and meta-analyses have suggested positive effects of music on cognitive function^25,27,78^. However, contrary to these findings, our study did not observe a significant effect of music therapy on global cognition compared to passive control conditions. This discrepancy may be attributed to sample heterogeneity, as previous studies focused on older people with moderate to severe cognitive impairment^25,27,78^. Music intervention appears to be more beneficial for individuals with cognitive impairment, as it primarily targets specific cognitive domains, particularly episodic memory, rather than global cognition. In daily life, the brain stores memories and emotions associated with significant episodes accompanied by music, which can trigger the retrieval of autobiographical memories^11,79^. Music may be particularly helpful for individuals with AD dementia who struggle with recalling salient autobiographical contents. However, the effect of improving global cognition in older adults with no or mild cognitive decline may not have reached statistical significance in our study.

Consistent with previous meta-analyses, our findings revealed that exercise intervention ameliorated global cognition in older adults, both with and without MCI. However, the relative effect size of exercise intervention ranked low on the list of interventions. This could be attributed to bias resulting from averaging the results of different exercise interventions. Multiple meta-analyses examining the effect of exercise intervention on global cognition in older adults with MCI, as well as in disease-free older adults, have found significant variations in effect sizes across studies^43,80–86^. The relationship between exercise intervention and global cognition may depend on the type of exercise being employed^87^. Previous studies have shown that different types of exercise can have varying effects on cognition, possibly due to different molecular mechanisms. For instance, the SMD for resistance exercise ranges from 0.41 to 0.71, while the SMD for aerobic exercise ranges from 0.13 to 0.58^84,86,88,89^. Therefore, it is important to consider the type of exercise when prescribing exercise intervention with the aim of preventing or decelerating cognitive deterioration.

Additionally, our subgroup analysis of the MCI group or healthy older group revealed some inconsistent results (Supplementary Figure S1). ACU intervention, CTI, and mind–body exercise were promising intervention types for preventing the decline of global cognition in both groups. However, the order of comparative effectiveness of these interventions varied across the two groups. Mind–body exercise was most likely to be the optimal intervention with a SUCRA value of 77.35% for the healthy older group. However, for the MCI group, ACU intervention had a slightly higher probability of being the most optimal intervention (SUCRA = 77.35%), higher than CTI (SUCRA = 77.21%) and mind–body exercise (SUCRA = 62.81%). This discrepancy might be attributed to the complexity of the mind–body exercise. Mind–body exercise combines various components including movement, breath control, and attention modulation. This characteristic might increase the complexity of implementing the intervention and reduce intervention fidelity (i.e., the consistency between plan and execution), especially for older adults with MCI^35,90^. Therefore, our results suggest that different treatments should be provided depending on the characteristics of the patient group.

In summary, our NMA found that mind–body exercise, ACU intervention, meditation, CTI, brain stimulation, and physical exercise were effective interventions for improving global cognition in older individuals, with a large effect size. However, music intervention did not show a significant effect in individuals with or without mild cognitive decline. Regarding the mechanism of action for each intervention, our study revealed that improvements in global cognition may occur through various pathways, such as memorizing movements and enhancing brain plasticity by reducing stress. Furthermore, the results of subgroup analyses demonstrated that intervention efficacy varied depending on the characteristics of the older adults. This suggests that healthcare professionals should consider patient characteristics when prescribing interventions to ensure the most effective treatment for their patients.

This study has some limitations. Firstly, there are some differences between the methods in this manuscript and those in the pre-registration. In the pre-registration, the original participants for the study were patients with dementia and MCI. However, for this study, dementia patients were excluded and people with normal cognitive function were included. As this study aimed to evaluate and compare the efficacies of nonpharmacological interventions alone, RCT studies involving both nonpharmacological and pharmacological interventions were excluded from the analyses. Most RCT studies of patients with dementia typically involve both types of interventions. Therefore, the studies on patients with dementia were excluded from this study. If the studies were collected according to the research steps initially proposed in the pre-registration, the sample size would be small, which could lead to an overestimated treatment efficacy^91^. Therefore, we broadened the sample group pool by including both individuals with normal function and patients with MCI. Secondly, the number of included studies, especially those investigating ACU or music interventions, was relatively small. Thirdly, although our review explored multiple nonpharmacological interventions for global cognition, we were unable to consider factors such as intervention length, duration, frequency, psychological status, current medication use, and participant sex ratios due to insufficient information. To identify the effects of these variables on global cognition, further NMAs will be needed using these variables as covariates. Fourthly, we specifically focused on older people with or without minor cognitive decline and excluded research involving those with other psychiatric or neurological disorders, which represent only a subset of the older population worldwide. Fifthly, we excluded studies that combined multiple interventions or interventions with pharmacological treatments due to limitations in statistical analysis. Finally, including studies utilizing various scales to measure global cognition may have introduced a potential limitation. However, previous studies have demonstrated a strong correlation among different measures of global cognition^92^.

Despite these limitations, our study demonstrated the efficacy of seven interventions and provided insights into the treatment mechanisms for improving global cognition in older adults with and without mild cognitive decline. Considering the concurrent use of medications, variations in drug metabolism, a higher risk of adverse effects, and the presence of comorbid medical conditions, this population may exhibit greater sensitivity to pharmacological interventions compared to younger individuals^93^. Based on our review, nonpharmacological interventions, particularly mind–body exercises, could be a potential strategy for enhancing and maintaining global cognition in the older population.

However, the generalizability of these findings and the ability to draw definitive conclusions regarding the efficacy of these interventions are limited. To gain a better understanding of how intervention methods or psychological variables impact global cognition, future studies should incorporate additional moderating variables in the design of RCTs, such as intervention length, duration, frequency, psychological status, current medication use, and participant sex ratios.

### Supplementary Information

Supplementary Information.

## Data Availability

The data supporting the findings of this study are available from the corresponding author upon reasonable request.

## Abbreviations

AD: Alzheimer’s disease
ADAS-Cog: Alzheimer’s Disease Assessment Scale-Cognitive subscale
CAMCOG: Cambridge Cognitive Examination
CENTRAL: Cochrane Central Register of Controlled Trials
CTI: Cognitive training intervention
MMSE: Mini-Mental State Examination
MCI: Mild cognitive impairment
MoCA: Montreal Cognitive Assessment
NMA: Network meta-analysis
PRISMA: Preferred Reporting Items for Systematic Reviews and Meta-Analyses
RBANS: Repeatable Battery for the Assessment of Neuropsychological Status
RCT: Randomized controlled trial
tDCS: Transcranial direct current stimulation
TMS: Transcranial magnetic stimulation
